# Postoperative outcomes after degenerative lumbar spine surgery in rheumatoid arthritis patients -a propensity score-matched analysis

**DOI:** 10.1186/s12891-022-05326-5

**Published:** 2022-04-22

**Authors:** So Kato, Hideki Nakamoto, Yoshitaka Matsubayashi, Yuki Taniguchi, Toru Doi, Yuichi Yoshida, Akiro Higashikawa, Yujiro Takeshita, Masayoshi Fukushima, Takashi Ono, Nobuhiro Hara, Rentaro Okazaki, Hiroki Iwai, Masahito Oshina, Shurei Sugita, Shima Hirai, Kazuhiro Masuda, Sakae Tanaka, Yasushi Oshima, University of Tokyo Spine Group, Yasushi Oshima, Naohiro Kawamura, Akiro Higashikawa, Nobuhiro Hara, Takashi Ono, Yujiro Takeshita, Yuki Taniguchi, Yoshitaka Matsubayashi, So Kato

**Affiliations:** 1 Department of Orthopaedic Surgery, the University of Tokyo, 7-3-1 Hongo, Bunkyo-ku, Tokyo, 113-8655 Japan; 2 Department of Spine and Orthopedic Surgery, Japanese Red Cross Medical Center, 4-1-22 Hiroo, Shibuya-ku, Tokyo, 150-8935 Japan; 3 Department of Orthopedic Surgery, Japan Organization of Occupational Health and Safety Kanto Rosai Hospital, Kizukisumiyoshi-cho, Nakahara-ku, Kawasaki, 211-8510 Japan; 4 Department of Orthopedic Surgery, Japan Organization of Occupational Health and Safety Yokohama Rosai Hospital, 3211 Kozukue-Chō, Kōhoku-Ku, Yokohama, 222-0036 Japan; 5 Spine Center, Toranomon Hospital, 2-2-2 Toranomon, Minato-ku, Tokyo, 105-8470 Japan; 6 Department of Spinal Surgery, Japan Community Health-Care Organization Tokyo Shinjuku Medical Center, 5-1 Tsukudo-cho, Shinjuku-ku, Tokyo, 162-8543 Japan; 7 Department of Orthopedic Surgery, Japanese Red Cross Musashino Hospital, 1-26-1 Kyonancho, Musashino, Tokyo, 180-8610 Japan; 8 Department of Orthopedic Surgery, Saitama Red Cross Hospital, 1-5 Shintoshin, Chuo-ku, Saitama, 330-8553 Japan; 9 Iwai Orthopaedic Medical Hospital, 8-17-2 Minamikoiwa Edogawa-ku, Tokyo, 133-0056 Japan; 10 Department of Orthopedic Surgery, NTT Medical Center Tokyo, 5-9-22 Higashi-Gotanda, Shinagawa-ku, Tokyo, 141-8625 Japan; 11 Department of Orthopedic Surgery, Tokyo Metropolitan Cancer and Infectious Diseases Center Komagome Hospital, 3-18-22 Honkomagome, Bunkyo-ku, Tokyo, 113-8677 Japan; 12 Department of Orthopedic Surgery, Sagamihara National Hospital, 18-1 Sakuradai, Minami-ku, Sagamihara, Kanagawa 252-0392 Japan; 13 Department of Orthopedic Surgery, Tokyo Metropolitan Tama Medical Center, 2-8-29 Musashidai, Fuchu, Tokyo, 183-8524 Japan

**Keywords:** Degenerative lumbar disease, Lumbar spinal stenosis, Rheumatoid arthritis, Posterior surgery, Patient-reported outcomes, Numerical rating scale, Short-form 12 physical component summary, EuroQOL, Oswestry Disability Index, Propensity score matching

## Abstract

**Background:**

Although treatment options for rheumatoid arthritis (RA) have evolved significantly since the introduction of biologic agents, degenerative lumbar disease in RA patients remains a major challenge. Well-controlled comparisons between RA patients and their non-RA counterparts have not yet been reported. The objective of the present study was to compare postoperative outcomes of lumbar spine surgery between RA and non-RA patients by a retrospective propensity score-matched analysis.

**Methods:**

Patients who underwent primary posterior spine surgery for degenerative lumbar disease in our prospective multicenter study group between 2017 and 2020 were enrolled. Demographic data including age, sex, body mass index (BMI), American Society of Anesthesiologists (ASA) physical status classification, diabetes mellitus, smoking, steroid usage, number of spinal levels involved, and preoperative patient-reported outcome (PRO) scores (numerical rating scale [NRS] for back pain and leg pain, Short Form-12 physical component summary [PCS], EuroQOL 5-dimension [EQ-5D], and Oswestry Disability Index [ODI]) were used to calculate a propensity score for RA diagnosis. One-to-one matching was performed and 1-year postoperative outcomes were compared between groups.

**Results:**

Among the 4567 patients included, 90 had RA (2.0%). RA patients in our cohort were more likely to be female, with lower BMI, higher ASA grade and lower current smoking rate than non-RA patients. Preoperative NRS scores for leg pain, PCS, EQ-5D, and ODI were worse in RA patients. Propensity score matching generated 61 pairs of RA and non-RA patients who underwent posterior lumbar surgery. After background adjustment, RA patients reported worse postoperative PCS (28.4 vs. 37.2, *p* = 0.008) and EQ-5D (0.640 vs. 0.738, *p* = 0.03), although these differences were not significant between RA and non-RA patients not on steroids.

**Conclusions:**

RA patients showed worse postoperative quality of life outcomes after posterior surgery for degenerative lumbar disease, while steroid-independent RA cases showed equivalent outcomes to non-RA patients.

## Background

Rheumatoid arthritis (RA) is a systemic inflammatory disease that involves multiple joints  [1]. Synovitis leading to joint destruction is well known to involve major weight-bearing joints such as the knees and ankles. RA also affects the spine by damaging the synovium in facet joints. Classically, atlantoaxial instability is one of the pathognomonic changes seen in RA patients  [2]. In recent years, treatment options for RA have significantly evolved. Disease-modifying antirheumatic agents including methotrexate (MTX) and biologics such as Tissue Necrosis Factor alpha (TNF-α) inhibitors and Janus kinase (JAK) inhibitors have drastically changed the prognosis of joint destruction  [3]. As for spinal pathology, atlantoaxial instability and subsequent basilar invagination are also known to be significantly suppressed following introduction of biologics  [4].

The lumbar spine is not exempt from the destruction seen in RA  [5, 6]. RA in the lumbar spine manifests as spinal canal stenosis as well as back pain and spondylolisthesis caused by joint instability  [7]. In particular, spondylolisthesis has been reported more frequently in RA patients than in the general population, possibly due to facet joint erosion  [8, 9]. Despite tremendous success in the treatment of cervical spine pathologies in RA, degenerative lumbar disease in RA remains a major challenge. Lumbar spondylopathy has also become a significant burden as a growing issue among RA patients with the improvements in activities of daily living seen in the era of biologics  [10].

In addition to the complexity of spinal pathology, RA patients also suffer a higher rate of complications such as vertebral fractures, surgical site infection and revisions required following spinal surgery  [11–13]. This has been attributed to impaired bone strength related to inflammation and steroid usage, immunosuppression due to RA treatment and progressive erosion of the facet joints. While outcomes in RA patients undergoing lumbar spinal surgery have been studied  [11, 12, 14–16], well-controlled comparisons between RA patients and their non-RA counterparts have yet to be reported. Complicating such comparisons is the fact that background health status and the pathologies found in lumbar spondylopathy differ between these two groups. The objective of the present study was to compare postoperative outcomes after lumbar spine surgery between RA and non-RA patients.

## Methods

### Patient sample and outcome measurements

Patients > 20 years old who underwent posterior spine surgery as a primary procedure for degenerative lumbar disease in our prospective multicenter study group (the University of Tokyo Spine Group) between 2017 and 2020 were enrolled. Demographic data including age, sex, body mass index (BMI), American Society of Anesthesiologists (ASA) physical status classification, diabetes mellitus, rheumatoid arthritis, smoking, steroid usage, and number of spinal levels involved were investigated. Surgical details including fusion level, operation time, and estimated blood loss were recorded. Patient-reported outcome (PRO) scores were also collected preoperatively by distributing questionnaires including a numerical rating scale (NRS) for back pain, NRS for leg pain, 12-item Short Form (SF-12) physical component summary (PCS)  [17], EuroQOL 5-dimension (EQ-5D)  [18], and Oswestry Disability Index (ODI)  [19]. Complications occurring within 30 days after surgery were recorded and categorized as neurological, surgical site infection, hematoma, implant-related, organ damage, respiratory, urinary tract infection, cardiovascular/cerebrovascular, in-hospital death, or other. All patients were encouraged to attend the 1-year follow-up appointment and to complete the same PRO questionnaires.

### Propensity score-matched analysis

To make comparisons between RA and non-RA patients adjusted for preoperative background factors, propensity score matching was performed. Propensity score-matched analysis is widely used in cohort studies to adjust for confounding biases  [20]. With this statistical approach, propensity scores estimate the probability of a patient being diagnosed with RA based on patient characteristics. Propensity scores were calculated from logistic regression models. In the present study, demographic data (age, sex, BMI, ASA classification, diabetes mellitus, smoking, and number of spinal levels involved) as well as preoperative PRO scores (NRS back pain, NRS leg pain, PCS, EQ-5D, and ODI) were used to calculate a propensity score for RA diagnosis. Fusion was not used as a variable because this was a resultant treatment option rather than a background factor discriminating RA and non-RA. Next, one-to-one matching was performed to match one patient with RA to another non-RA patient with the same propensity score, representing comparable background characteristics. The pairing was performed with the caliper tolerance of 20% of standard deviation of propensity score, and a random selection was made among the patients with the same propensity score. RA and non-RA patients were gathered to form two groups with similar backgrounds for comparison. Matched RA patients were further investigated for pre-operative serum C-reactive protein (CRP), steroid dosage, MTX and biologic use. One-year postoperative PRO scores and 30-day complication rates were compared between groups.

### Statistical analyses

All analyses were carried out using the IBM SPSS Statistics version 26 (IBM Corp., Armonk, NY). To analyze differences between groups, a paired *t*-test, Wilcoxon signed-rank test, or Mann–Whitney U-test was used for continuous variables and McNemar’s test or Chi-square test was used for categorical variables. For comparisons among three groups, the Kruskal–Wallis test with Dunn-Bonferroni post-hoc testing was used. For all statistical tests, values of *p* < 0.05 were considered significant.

## Results

### Demographics

Among the 4567 patients included, 90 had RA (2.0%). Demographic data in each group are summarized in Table 1. Compared to non-RA patients, RA patients in our cohort were more likely to be female (80.0% vs. 41.7%, *p* < 0.001), with lower BMI (23.2 kg/m^2^ vs. 24.4 kg/m^2^, *p* = 0.002), higher ASA classification (*p* = 0.01) and lower current smoking rate (2.2% vs. 10.6%, *p* = 0.01). Among patients who successfully completed preoperative PRO questionnaires, NRS for leg pain (7.3 vs. 6.6, *p* = 0.03), PCS (19.0 vs. 26.1, *p* < 0.001), EQ-5D (0.514 vs. 0.558, *p* = 0.01), and ODI (51.1 vs. 42.1, *p* < 0.001) were all worse in RA patients.

**Table 1:** Demographic data

	Total	RA	Non-RA	p
n	4567	90	4477	
Age (yrs, mean [SD])	70.3 (10.4)	72.4 (8.0)	70.2 (10.5)	0.09
Sex (male, %)	57.5	20.0	58.3	< 0.001
BMI (kg/m^2^, mean [SD])	24.4 (3.7)	23.2 (3.6)	24.4 (3.7)	0.002
ASA grade (median [range])	2 (1—4)	2 (1—3)	2 (1—4)	0.01
Diabetes mellitus (%)	17.0	20.0	17.0	0.45
Current smoker (%)	10.4	2.2	10.6	0.01
Number of spinal levels involved	2.7 (0.9)	2.8 (0.9)	2.7 (0.9)	0.54
Fusion (%)	34.1	48.9	33.8	0.003
Preoperative PRO (mean [SD])				
Back pain (NRS)	5.5 (3.0)	6.0 (3.2)	5.5 (3.0)	0.14
Leg pain (NRS)	6.6 (2.8)	7.3 (2.6)	6.6 (2.8)	0.03
SF-12 (PCS)	26.0 (14.3)	19.0 (13.2)	26.1 (14.3)	< 0.001
EQ-5D	0.557 (0.157)	0.514 (0.171)	0.558 (0.157)	0.01
ODI	42.2 (17.8)	51.1 (18.6)	42.1 (17.7)	< 0.001

*SD* Standard deviation, *BMI* Body mass index, *ASA* American Society of Anesthesiologists Classification, *PRO* Patient-reported outcome, *NRS* Numeric rated scale, *PCS* Physical component summary, *EQ-5D* EuroQOL 5-dimension, *ODI* Oswestry Disability Index, *RA* Rheumatoid arthritis

Values are shown in mean with standard deviation or percentage, with exception of ASA grade shown as median and range

No significant differences were seen in the number of spinal levels involved, but fusion surgery was more common in the RA group (48.9% vs. 33.8%, *p* = 0.003). Operation time was longer (162 min vs. 140 min, *p* = 0.01) and estimated blood loss was greater (254 vs. 154 mL, *p* < 0.001) in the RA group.

A total of 2394 patients (52.4%) completed 1-year postoperative PRO questionnaires, enabling further analysis. Table 2 shows the results for PRO scores with intergroup comparisons. All PRO scores investigated in the present study showed significant postoperative improvement compared to preoperative scores (*p* < 0.001) for the entire cohort. Comparisons between RA and non-RA patients revealed that postoperative NRS for leg pain, PCS, EQ-5D, and ODI were significantly worse in RA patients. Thirty-day complication rates were higher in the RA group (11.1%) than in the non-RA group (4.5%, *p* = 0.003), with urinary tract infection as the most frequent complication among RA patients (3.3%).

**Table 2:** Postoperative outcomes in RA and non-RA patients

	Total	RA	Non-RA	p
n	2394	46	2348	
Back pain (NRS)	2.8 (2.7)	3.0 (2.7)	2.9 (2.7)	0.76
Leg pain (NRS)	3.1 (3.0)	4.6 (3.1)	3.1 (3.0)	0.001
SF-12 (PCS)	38.2 (14.8)	28.4 (14.5)	38.4 (14.8)	< 0.001
EQ-5D	0.742 (0.188)	0.634 (0.214)	0.744 (0.187)	< 0.001
ODI	20.5 (18.2)	34.1 (23.0)	20.2 (18.0)	< 0.001

*NRS* Numeric rated scale, *PCS* Physical component summary, *EQ-5D* EuroQOL 5-dimension, *ODI* Oswestry Disability Index, *RA* Rheumatoid arthritis

Values are shown in mean with standard deviation in parentheses

### Propensity score matching

Propensity score matching generated 61 pairs of RA and non-RA patients who underwent posterior lumbar surgery. As expected, no differences were evident between groups for any of the factors included in propensity score calculation, and the two groups showed statistically identical baseline characteristics (Table 3). Fusion surgery represented approximately half of surgeries in both groups (49% vs. 46%, *p* = 0.72).

**Table 3:** Comparison of background characteristics between propensity score-matched groups

	RA	Non-RA	p
Age (yrs, mean [SD])	72.3 (7.8)	70.3 (11.0)	0.53
Sex (male, %)	18.0	18.0	> 0.99
BMI (kg/m2, mean [SD])	23.5 (3.6)	23.6 (3.9)	0.96
ASA grade (median, range)	2 (1—3)	2 (1—3)	0.78
Diabetes mellitus (%)	25.0	26.0	0.84
Current smoker (%)	1.6	1.6	> 0.99
Number of spinal levels involved	2.8 (0.8)	2.7 (0.8)	0.39
Fusion (%)	49	46	0.72
Preoperative PRO (mean [SD])			
Back pain (NRS)	6.2 (3.1)	5.8 (3.3)	0.55
Leg pain (NRS)	7.4 (2.4)	7.7 (2.5)	0.26
SF-12 (PCS)	18.7 (13.0)	21.6 (15.2)	0.18
EQ-5D	0.509 (0.178)	0.520 (0.185)	0.62
ODI	52.2 (19.3)	50.2 (16.9)	0.43

*SD* Standard deviation, *BMI* Body mass index, *ASA* American Society of Anesthesiologists Classification, *PRO* Patient-reported outcome, *NRS* Numeric rated scale, *PCS* Physical component summary, *EQ-5D* EuroQOL 5-dimension, *ODI* Oswestry Disability Index, *RA* Rheumatoid arthritis

Values are shown in mean with standard deviation or percentage, with exception of ASA grade shown as median and range

In this matched cohort, a total of 40 pairs (65.6%) completed 1-year postoperative PRO questionnaires, enabling further analyses. Although similar interventions were performed for the two groups of patients with similar backgrounds, RA patients still reported worse postoperative PCS (28.4 vs. 37.2, *p* = 0.008) and EQ-5D (0.640 vs. 0.738, *p* = 0.03) than non-RA patients (Table 4), although 30-day complications rates did not differ significantly between RA and non-RA groups (11.5% vs. 3.3%, *p* = 0.08).

**Table 4:** Comparison of postoperative outcomes between propensity score-matched RA and non-RA groups

	RA	Non-RA	p
n	40	40	
Back pain (NRS)	3.2 (2.7)	3.5 (2.7)	0.42
Leg pain (NRS)	4.7 (3.1)	3.8 (3.2)	0.20
SF-12 (PCS)	28.4 (15.2)	37.2 (14.4)	0.008
EQ-5D	0.640 (0.225)	0.738 (0.158)	0.03
ODI	32.2 (19.9)	24.6 (17.7)	0.07

*NRS* Numeric rated scale, *PCS* Physical component summary, *EQ-5D* EuroQOL 5-dimension, *ODI* Oswestry Disability Index, *RA* Rheumatoid arthritis

Values are shown in mean with standard deviation in parentheses

### Impact of steroid usage on postoperative outcomes (post-hoc analysis)

**Fig. 1:**
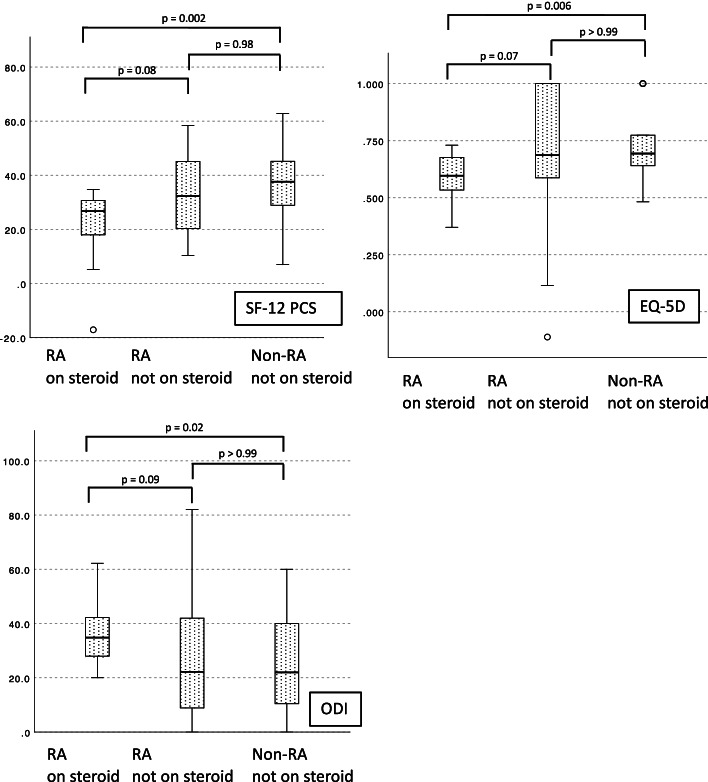
Comparison of postoperative outcomes among propensity score-matched RA patients on steroids, RA patients not on steroids, and non-RA patients not on steroids. PCS: physical component summary, EQ-5D: EuroQOL 5-dimension, ODI: Oswestry Disability Index, RA: rheumatoid arthritis

Approximately half of the RA group (18 out of 40 patients) were steroid users, while only one patient in the non-RA group was using steroids (*p* < 0.001). Among RA patients on steroid, the dosage ranged from 0 to 10 prednisolone mg equivalents per day with a mean 2.2 mg/day. MTX usage and biologic usage were not different between RA patients with and without steroid (MTX: 50% vs. 50%, *p* > 0.99, biologics: 28% vs. 14%, *p* = 0.43), but preoperative serum CRP were significantly higher in RA patients on steroid (mean 0.80 vs. 0.51 mg/dL, *p* = 0.03). To clarify the impact of steroid usage on postoperative outcomes, further analysis was conducted to compare outcomes among non-steroid users without RA (*n* = 39), non-steroid users with RA (*n* = 22), and steroid users with RA (*n* = 18). Kruskal–Wallis testing revealed significant differences among the three groups for PCS (*p* = 0.003), EQ-5D (*p* = 0.007), and ODI (*p* = 0.02), despite a lack of significant differences in back or leg pain (*p* = 0.15 and 0.25). Comparisons of PRO scores are summarized in Fig. 1. According to post-hoc analyses, steroid-dependent RA patients showed significantly inferior results to non-RA patients in PCS (mean: 23.0 vs. 37.3, *p* = 0.002), EQ-5D (0.580 vs. 0.737, *p* = 0.006), and ODI (37.5 vs. 24.2, *p* = 0.02), whereas steroid-independent RA patients showed comparable results to non-RA patients. Among the steroid-dependent RA patients, those with daily steroid dosage equivalent to 5 mg of prednisolone or more tended to show the lowest quality of life scores with mean PCS of 18.5, mean EQ-5D of 0.582 and mean ODI of 39.7. 30-day complications rates did not differ significantly among the three groups (17% in steroid-dependent RA patients, 18% in steroid-independent RA patients, 3% in non-RA patients, *p* = 0.09).

## Discussion

The present study used propensity score-matched analyses to elucidate the differences in outcomes following lumbar spinal surgery between RA and non-RA patients. This controlled comparative study minimized the risk of biases in terms of patient demographics, allowing us to elucidate the true impact of RA as a diagnosis on postoperative outcomes following lumbar surgery. Our results revealed that RA patients showed worse SF-12 PCS and EQ-5D than the non-RA matched cohort, indicating that RA negatively affected postoperative outcomes. However, steroid-independent RA patients showed comparable outcomes to non-RA patients.

Very few previous studies have reported on the relative equivalence of postoperative outcomes for RA and non-RA patients. Crawford et al. first reported that clinical outcomes after posterolateral lumbar fusion as graded using the Herkowitz and Kurtz scale [21] (excellent, good, fair, and poor) were similar between 19 RA patients and 19 age-, sex-, smoking status-, and spinal level-matched non-RA patients, although RA patients displayed a slightly higher complication rate due to osteopenia and immunosuppression  [11]. Gulati et al. showed more detailed outcome measures, including ODI, EQ-5D, NRS for back pain, and NRS for leg pain at 1 year after lumbar decompression surgery, finding no differences between 37 RA patients and 1396 non-RA patients, although the two cohorts were not matched and background characteristics differed  [14]. Gulati et al. denied any increased risk of complications in the RA group. In contrast, Kang et al. more recently argued that 40 RA patients displayed lower ODI scores at 1 and 2 years after posterolateral lumbar fusion, compared to a total of 134 age-, sex-, bone mineral density-, smoking-, diabetes-, and spinal level-matched non-RA patients  [16]. In summary, the conclusions drawn from previous studies have been inconsistent, with some including non-matched cohorts or patients treated using outdated surgical strategies.

Kang et al. speculated in their report showing poorer improvement in RA than in non-RA patients that multiple joint involvement in RA lead to increased overall disability  [16]. It is true that quality of life in RA patients is inherently impaired due to the destruction of multiple joints along with the associated chronic pain [22]. Postoperative health conditions are thus inevitably affected in a negative manner compared to non-RA patients, and fair comparison between the two groups has remained a huge challenge. The novelty of the present study lies in the meticulous propensity score-matching process incorporating preoperative PRO measurements. Through the statistical adjustment of background factors between RA and non-RA patients, this baseline impairment was already accounted for before the comparisons. The present study achieved inclusion of the largest number of matched pairs with the most statistically robust matching method in the literature. In the present propensity-score matched analysis, our results supported the findings of Kang et al., [16] suggesting unfavorable postoperative outcomes in RA patients even after adjustment for background. Interestingly, however, these differences were missing in comparisons among non-steroid users. These results implicated that poorer outcomes in RA patients were mainly due to steroid-related complications. These quite encouraging findings for both RA patients and spinal surgeons can be explained by two major hypotheses. First, the side effects of steroid treatment appear closely related to possible complications that could occur after lumbar spinal surgery and thereby negatively affect patient outcomes. Immunosuppression can lead to a higher chance of surgical site infection as well as other types of infectious complication, such as urinary tract infection and aspiration pneumonia  [23]. Osteopenia and osteoporosis can result in vertebral fractures in adjacent segments and instrument failure due to screw loosening  [12]. Steroid usage has been part of the classic presentation of RA patients and places significant burdens on surgical outcomes in general. Mitsuyama et al. also classically reported the pitfalls in surgical management of lumbar spinal canal stenosis in RA patients lie in the higher risk of infection, instrumentation failure and vertebral fracture showing the post-operative results of their 11 out of 12 patients being on steroid  [24]. Second, as treatment options have markedly increased with the introduction of biologics, steroid usage might be interpreted as a surrogate marker of suboptimal RA control  [25]. This may not be the case for all RA patients on steroids, but steroid-independent RA patients as a group might have achieved better disease control, and thus show a lower likelihood of ongoing joint destruction and instability.

Several limitations to the present study must be kept in mind when interpreting these findings. First, the database used in the analysis was obtained retrospectively and some pertinent information related to treatments received by patients was not obtainable. For instance, serological and/or physical proofs of RA control, including serum erythrocyte sedimentation rate and Disease Activity Score in 28 joints, were also not investigated. Therefore, even though RA patients did show inferior postoperative outcomes, the explanations for these findings remain speculative, while RA patients on steroid, who were particularly associated with poor outcomes, showed higher serum CRP indicating suboptimal control of systemic inflammation. In particular, the effects of RA control or treatment options on postoperative outcomes need to be validated in future studies. A second limitation was the percentage of patients who provided completed questionnaires at 1 year postoperatively. Although loss to follow-up is an unavoidable problem in surveillance-based multicenter studies, potentially leading to selection bias, outcomes in non-responders may not be inferior to those of responders  [26]. Lastly, the present study focused on mid-term postoperative results and long-term outcomes with more than 2 years of follow-up are yet to be elucidated. A follow-up period of 1 year has been considered appropriate to analyze postoperative results following decompression, as Gulati et al. reported with a similar study design  [14], but instrumentation-related long-term complications such pseudarthrosis and adjacent segment disease ideally need to be investigated for 2 years  [16]. Further studies are warranted to elucidate whether RA or its control affect postoperative outcomes over the long term.

## Conclusions

In conclusion, a diagnosis of RA was associated with worse postoperative outcomes after posterior surgery for degenerative lumbar disease compared with propensity score-matched non-RA patients. However, steroid-independent RA cases showed comparable outcomes to steroid-independent non-RA patients. Contemporary well-controlled RA patients warrant a reconsideration of risk assessment for spinal surgery, as the previous impression of a high-risk profile may no longer be entirely appropriate.

## Data Availability

The datasets used and/or analysed during the current study are available from the corresponding author on reasonable request.

## Abbreviations

RA: Rheumatoid arthritis
BMI: Body mass index
ASA: American Society of Anesthesiologists
PRO: Preoperative patient-reported outcome
NRS: Numerical rating scale
SF-12: Short Form-12
PCS: Physical component summary
EQ-5D: EuroQOL 5-dimension
ODI: Oswestry Disability Index
TNF-α: Tissue Necrosis Factor alpha
JAK: Janus Kinase
